# Impact of systemic targeted agents on the clinical outcomes of patients with brain metastases

**DOI:** 10.18632/oncotarget.4153

**Published:** 2015-06-01

**Authors:** Adam G. Johnson, Jimmy Ruiz, Ryan Hughes, Brandi R. Page, Scott Isom, John T. Lucas, Emory R. McTyre, Kristin W. Houseknecht, Diandra N. Ayala-Peacock, Daniel J. Bourland, William H. Hinson, Adrian W. Laxton, Stephen B. Tatter, Waldemar Debinski, Kounosuke Watabe, Michael D. Chan

**Affiliations:** ^1^ Department of Radiation Oncology, Wake Forest School of Medicine, Winston-Salem, NC, USA; ^2^ Department of Medicine, Section on Hematology and Oncology, Wake Forest School of Medicine, Winston-Salem, NC, USA; ^3^ W.G. (Bill) Hefner Veteran Administration Medical Center, Cancer Center, Salisbury, NC, USA; ^4^ Department of Biostatistical Sciences, Wake Forest School of Medicine, Winston-Salem, NC, USA; ^5^ Department of Neurosurgery, Wake Forest School of Medicine, Winston-Salem, NC, USA; ^6^ Brain Tumor Center of Excellence, Wake Forest School of Medicine, Winston-Salem, NC, USA; ^7^ Department of Cancer Biology, Wake Forest School of Medicine, Winston-Salem, NC, USA

**Keywords:** targeted agents, stereotactic radiosurgery, brain metastases, chemotherapy

## Abstract

**Background:**

To determine the clinical benefits of systemic targeted agents across multiple histologies after stereotactic radiosurgery (SRS) for brain metastases.

**Methods:**

Between 2000 and 2013, 737 patients underwent upfront SRS for brain metastases. Patients were stratified by whether or not they received targeted agents with SRS. 167 (23%) received targeted agents compared to 570 (77%) that received other available treatment options. Time to event data were summarized using Kaplan-Meier plots, and the log rank test was used to determine statistical differences between groups.

**Results:**

Patients who received SRS with targeted agents vs those that did not had improved overall survival (65% vs. 30% at 12 months, *p* < 0.0001), improved freedom from local failure (94% vs 90% at 12 months, *p* = 0.06), improved distant failure-free survival (32% vs. 18% at 12 months, *p* = 0.0001) and improved freedom from whole brain radiation (88% vs. 77% at 12 months, *p* = 0.03). Improvement in freedom from local failure was driven by improvements seen in breast cancer (100% vs 92% at 12 months, *p* < 0.01), and renal cell cancer (100% vs 88%, *p* = 0.04). Multivariate analysis revealed that use of targeted agents improved all cause mortality (HR = 0.6, *p* < 0.0001).

**Conclusions:**

Targeted agent use with SRS appears to improve survival and intracranial outcomes.

## INTRODUCTION

The management options for patients with brain metastases have improved significantly over time due to effective methods for earlier detection [1], better brain-directed therapies such as combined modality therapies [2], and improvements in systemic chemotherapy [3, 4]. While a number of clinical trials have assessed the role of specific systemic agents for select populations of patients with brain metastases [5, 6], it remains unclear to what degree targeted systemic agents have affected the brain metastasis population as a whole. One population of particular interest is patients who receive stereotactic radiosurgery (SRS) as these patients are selected to have a limited burden of disease and a longer life expectancy. At this time, it is unclear whether the development of newer systemic agents, such as targeted agents, has improved clinical endpoints after SRS for the brain metastasis population as a whole, though evidence has emerged that certain subpopulations may benefit.

One population for which the use of targeted agents has affected clinical outcomes for brain metastases after SRS is patients with renal cell carcinoma. A recent series has shown that targeted agents not only improve overall survival in patients receiving SRS, but also improve upon the local efficacy of SRS on brain metastases [3]. Over the past decade, the use of targeted agents has proliferated for renal cell carcinoma [7–9], breast cancer [10], lung cancer [11], and melanoma [12] with improvements seen in overall survival in patients with metastatic disease. Given these benefits in metastatic disease, one question that emerges is whether or not targeted agent use affects brain metastasis outcomes.

The goal of the current study was to assess whether the promising interactions seen in the renal cell carcinoma population between targeted agents and SRS also exists with other primary tumors that metastasize to the brain. The current series represents one of the largest single institution series of patients treated with SRS for brain metastases. The study particularly aimed to assess the effect of targeted agent therapy on overall survival, local control, and the likelihood of distant brain failure in patients who received SRS for brain metastases, and whether these effects are primary tumor type or histology-specific.

## RESULTS

### Patient demographics

We identified 737 patients with brain metastases between January 2000 and December 2013. In total, 248 (33%) received targeted agents ever and 489 (67%) did not. A total of 167 patients (23%) received targeted agents either concurrently or within 30 days of SRS. Of these patients, 38 of 102 (37%) breast cancer, 10 of 40 colorectal cancer (25%), 70 of 364 (19%) lung cancer, 20 of 117 (17%) melanoma and 24 of 68 renal cell cancer patients (35%) received targeted agents within 30 days of SRS. Patients receiving targeted agents within 30 days of SRS had a younger age (median 58 vs 63 years, *p* = 0.002) and greater disease burden (43% vs. 32% widespread disease, *p* = 0.02) than those who did not. By the time of our analysis, 632 patients (86%) had died.

### Overall survival

**Figure 1:**
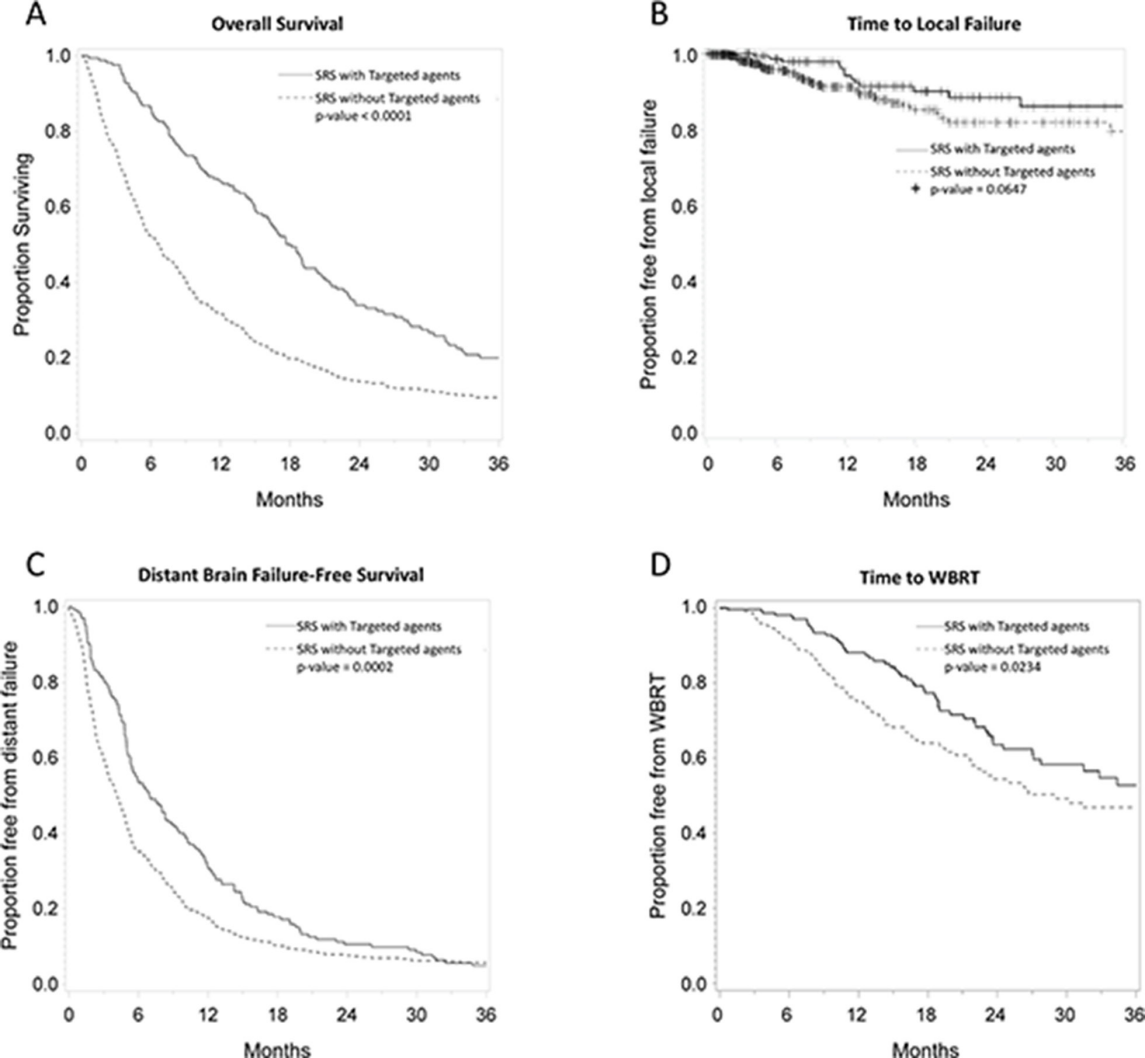
**Kaplan Meier plots comparing patients who received targeted agents vs. those who did not in terms of** overall survival **A.** freedom from local failure **B.** freedom from distant failure **C.** and freedom from salvage WBRT **D.**

Patients who received targeted agents within 30 days of SRS had a significant improvement in overall survival (Figure 1). The median overall survival was 7 months for the non-targeted agent use group and 18 months for the targeted agent group. Overall survival for targeted agent vs. non-targeted agent use groups was 90% vs. 55% at 6 months, 65% vs. 30% at 12 months, and 35% vs. 15% at 24 months (log rank *p* < 0.0001). There was no difference in neurologic death between patients receiving targeted agents within 30 days of SRS and those who did not (32% vs. 30%, respectively).

### Patterns of failure

Freedom from local failure for targeted agent vs. non-targeted agent use groups was 99% vs. 96% at 6 months, 94% vs. 91% at 12 months, and 88% vs. 84% at 24 months (log rank *p* = 0.06) (Figure 1). Distant brain failure-free survival for targeted agent vs. non-targeted agent use groups was 55% vs. 35% at 6 months, 32% vs. 18% at 12 months, and 10% vs. 8% at 24 months (log rank *p* = 0.0001). Time to salvage WBRT was 98% vs. 92% at 6 months, 88% vs. 77% at 12 months, and 64% vs. 54% at 24 months (log rank *p* = 0.03).

### Primary tumor-specific outcomes

**Figure 2:**
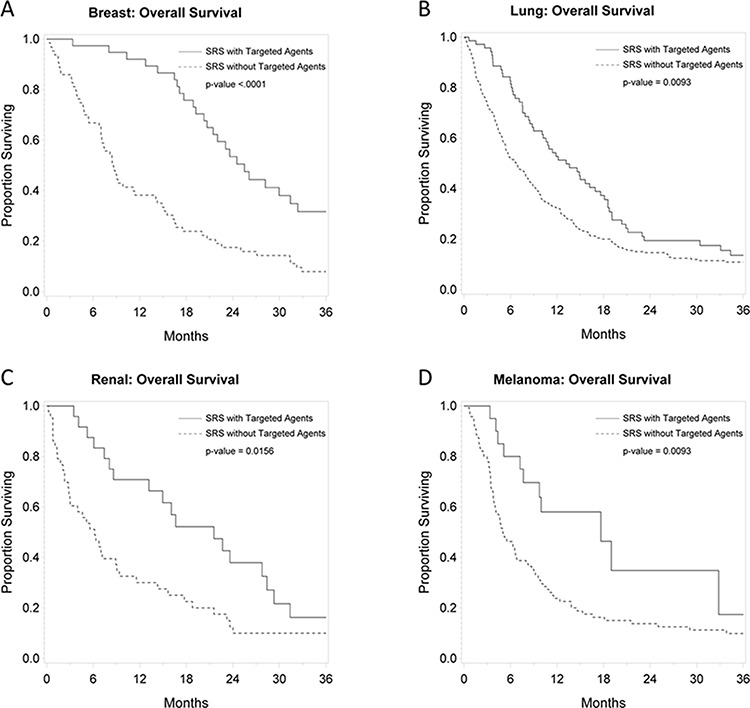
**Primary tumor-specific Kaplan Meier plots for overall survival comparing patients who received targeted agents vs. those who did not receive targeted agents concurrently or soon after SRS for** breast cancer **A.** lung cancer **B.** renal cell cancer **C.** and melanoma **D.**

**Figure 3:**
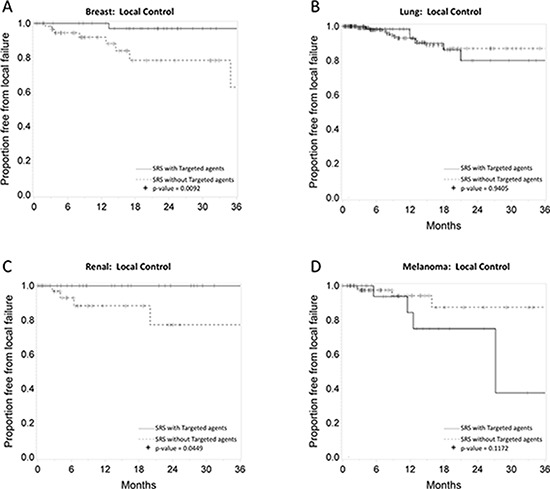
**Primary tumor-specific Kaplan Meier plots for freedom from local failure comparing patients who received targeted agents vs. those who did not receive targeted agents concurrently or soon after SRS for** breast cancer **A.** lung cancer **B.** renal cell cancer **C.** and melanoma **D.**

**Figure 4:**
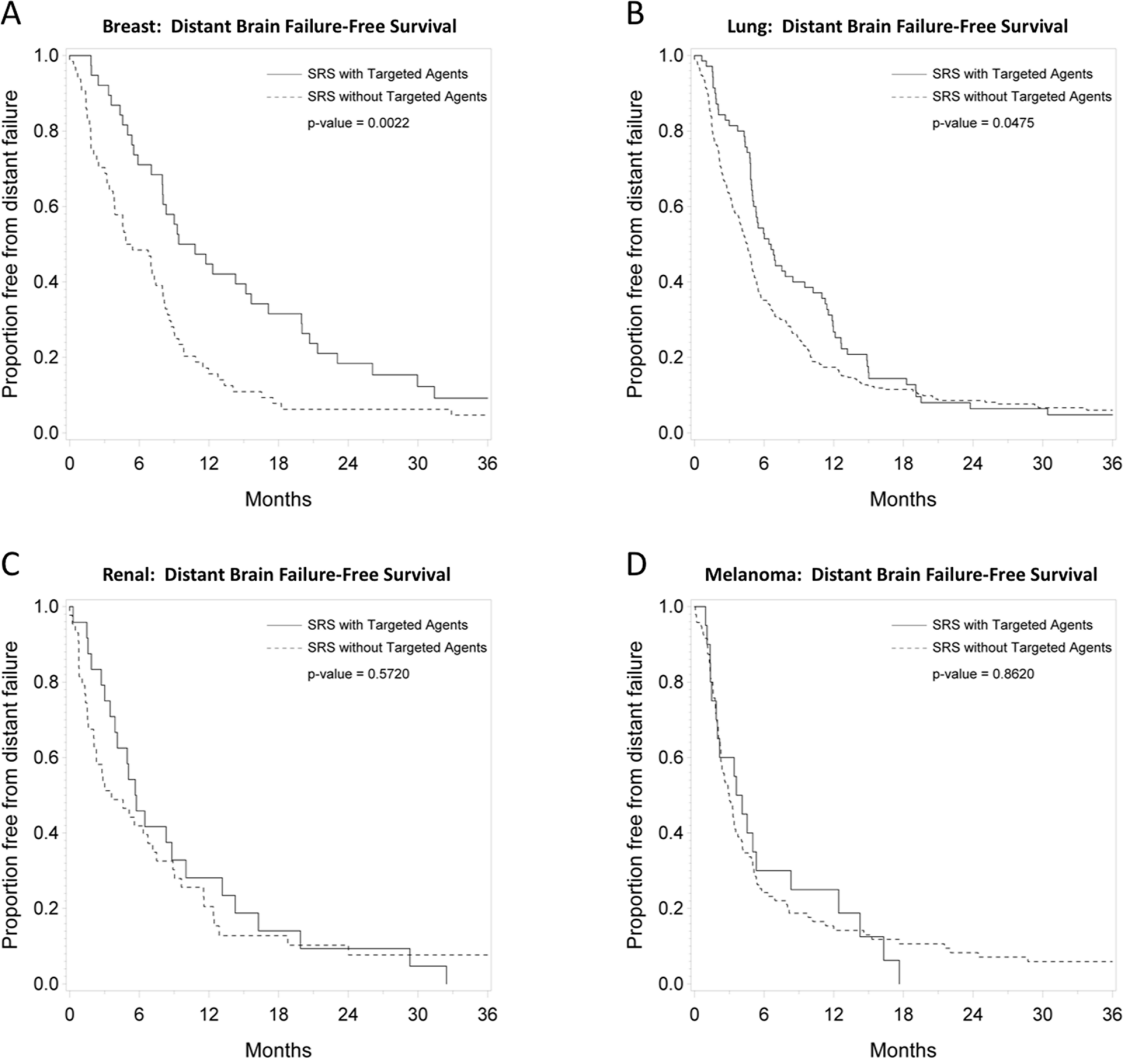
**Primary tumor-specific Kaplan Meier plots for distant failure-free survival comparing patients who received targeted agents vs. those who did not receive targeted agents concurrently or soon after SRS for** breast cancer **A.** lung cancer **B.** renal cell cancer **C.** and melanoma **D.**

Figures 2, 3, and 4 depict primary tumor-specific rates of overall survival, freedom from local failure, and distant brain failure-free survival for patients receiving targeted agents vs those that did not. For breast cancer brain metastases, patients receiving targeted agents experienced improved overall survival (median 24 months vs. 9 months, *p* < 0.0001), freedom from local failure (*p* < 0.01), and distant brain failure-free survival (median 10 vs. 5 months, *p* = 0.002). For brain metastases from non-small cell lung cancer, Kaplan Meier analysis revealed that patients receiving targeted agents experienced improved overall survival (median 13 vs. 7 months, *p* = 0.01) and freedom from distant brain failure-free survival (median 7 vs. 5 months, *p* = 0.048). For renal cell cancer brain metastases, patients receiving targeted agents experienced improved survival (21 vs. 6 months, *p* = 0.016), freedom from local failure (*p* = 0.04), but no significant improvement in distant brain failure-free survival rate (5 months for each group). For melanoma brain metastases, patients receiving targeted agents experienced improved survival (18 vs. 5 months, *p* = 0.009), but no significant improvement in rate of local failure (*p* = 0.1) or distant failure rate (4 months for each group). For brain metastases from colorectal cancers, patients receiving targeted agents did not experience any statistically significant differences in overall survival, local failure, or distant failure rates.

### Multivariate analysis of all-cause mortality

Results of multivariate analysis are shown in Table 1. Post-SRS targeted agent use was associated with significantly decreased hazard for all-cause mortality (*p* < 0.0001; HR 0.6; 95% CI 0.5–0.7). Increasing numbers of intracranial metastases relative to a solitary metastasis was associated with significantly increased hazard for all-cause mortality. This hazard increase was significant when comparing a solitary metastases with three (*p* = 0.01; HR 1.4; 95% CI 1.1–1.8), and four or more metastases (*p* < 0.001; HR 1.6; 95% CI 1.2–2.2). Oligometastatic disease relative to no evidence of disease, widespread disease relative to no evidence of disease, and progressive disease relative to stable disease were all associated with a significantly increased hazard for all-cause mortality (*p* = 0.04, HR 1.3, 95% CI 1.0–1.7; *p* = 0.005, HR 1.5, 95% CI 1.1–2.0; *p* < 0.0001, HR 1.7, 95% CI 1.4–2.0).

**Table 1:** Multivariate cox proportional hazards model for overall survival ^a^

Covariate	HR	95% CI	*P*
Age, 10 yr increase	1.1	1.0, 1.2	0.0592
GK Treatment year (1 year increase after the year 2000)	1.0	1.0, 1.0	0.8778
Gender: Women vs. men	0.9	0.8, 1.1	0.2291
DS-GPA, 1 unit increase	0.9	0.8, 1.0	0.0712
Number of courses of chemo			
1 vs. 0	1.2	1.0, 1.6	0.1094
2 vs. 0	1.0	0.7, 1.5	0.9016
3 vs. 0	1.1	0.8, 1.7	0.5055
4+ vs. 0	1.4	0.9, 2.2	0.0956
Number of intracranial metastasis			
2 vs. 1	1.2	1.0, 1.5	0.0621
3 vs. 1	1.4	1.1, 1.8	0.0144
4+ vs. 1	1.6	1.2, 2.2	0.0009
Disease Burden			
Oligometastatic vs. none	1.3	1.0, 1.7	0.0461
Widespread vs. none	1.5	1.1, 2.0	0.0052
Unknown vs. none	1.0	0.7, 1.6	0.9190
Systemic Disease			
Progressive vs. stable	1.7	1.4, 2.0	< 0.0001
Unknown vs. stable	1.2	0.9, 1.6	0.3151
Symptoms: yes vs. no	1.7	1.4, 2.1	< 0.0001
Neurosurgery: yes vs. no	0.6	0.5, 0.7	< 0.0001
Targeted agent: yes vs. no	0.6	0.5, 0.7	< 0.0001

Abbreviations: HR, Hazard ratio; GK, Gamma Knife; DS-GPA, disease-specific Graded Prognostic Assessment; WBRT, Whole Brain Radiotherapy; SRS, Stereotactic Radiosurgery.

a Cox model stratified by WBRT (yes/no) and repeat SRS (yes/no)

## DISCUSSION

Targeted agents represent a broad class of systemic therapies that inhibit cancer cells by specifically blocking molecular pathways that lead to tumor growth [16]. Because of the specificity of targeted agents and the multiple pathways of carcinogenesis across tumor types, these agents are generally specific to a tumor subtype and/or histological subtype. In the current study, the use of targeted systemic therapy demonstrated a survival advantage in patients with brain metastases across multiple primary tumor types. While a survival advantage is expected, the improvement in distant brain failure-free survival, time to WBRT and particularly in local control has wide clinical implications for the management of brain metastases.

Development of new metastases after SRS and the subsequent salvage treatments can significantly raise the cost of managing a patient with brain metastasis [17]. This is particularly true in patients that require early WBRT since the major advantage of SRS is the ability to avoid WBRT-related toxicty [18]. Several efforts are currently underway to improve patient selection for SRS by attempting to predict which patients will suffer rapid development of new metastases and require early WBRT [19]. Given the results of the current study, it would appear that patients receiving systemic targeted agents are not only less likely to require WBRT, but are also more likely to survive long enough to benefit from the cognitive toxicity-sparing effects of SRS. As such, practitioners may be able to use this factor as part of the selection criteria for patients to receive SRS instead of WBRT.

Morbidity and death from local failure after SRS is an important endpoint. Several series have suggested that some subgroups of brain metastases benefit from concurrent or post-SRS systemic therapy by improving local control following SRS [3, 20]. The current series confirms these previous findings across multiple primary cancers including lung cancer, breast cancer, melanoma, and renal cell cancer, the first time such a finding has been made over multiple histologies. A longstanding oncologic dogma has been that most systemic agents, whether targeted or cytotoxic, do not cross the blood brain barrier at a concentration high enough to lead to a sufficient response for brain metastases. Based on the current study, it appears that systemically administered targeted agents can improve local control of SRS when administered concurrently or soon after SRS. This benefit in local control is analogous to how concurrent chemotherapy increases local control of radiotherapy for patients with such cancers as head and neck cancer [21] or cervical cancer [22] where no blood brain barrier is involved. The possible implications of these findings are that when local control is suboptimal, such as in the case of large metastatic brain tumors, there may be a benefit to post-SRS targeted therapy, even in the absence of active extracranial disease.

There are several mechanisms by which the combination of SRS and targeted agents lead to improved local control in spite of questionable blood brain barrier penetration. First of all, it is thought that one of the reasons that SRS has improved efficacy over fractionated radiation is that it also targets the tumor vasculature [23]. Such targeting may disrupt the blood brain barrier so that drug can penetrate. This theory could explain the several negative prospective studies using targeted agents either as monotherapy [6] or in conjunction with conventionally fractionated whole brain radiotherapy [24]. Another hypothesis is that the increased anti-cancer activity of the combination of SRS and targeted agents may be due to the highly-specific targeting of driver mutations that may not require a high concentration to cause radiosensitization. Finally, a proportion of brain metastases may have pre-existing disruption of the blood brain barrier caused by the cancer so that penetration of targeted agents into brain metastases is possible [25]. Our data indeed showed that while the rate of local failure was significantly improved by the combination of SRS and targeted agents in most cancers, the distant failure rate did not improve universally. Our observations may partly support these hypotheses.

From the current study, it appears that the improvements in brain metastasis outcomes brought about by targeted agents are dependent upon the primary cancer subtype. Breast cancer and renal cell cancer appear to have the greatest benefits in survival compared to other primary cancer subtypes. Median overall survival in each of these populations was greater than 20 months. With regards to local brain failure, only breast cancers and renal cell cancers experienced statistically significant improvements. These primary cancer-specific outcomes imply that clinical studies for the use of targeted agents may require the molecular classification of the tumor type. With molecular classification, specific populations that benefit may then be identified. Another question for future trials will be the question of whether maintenance systemic therapy, given the improvement in distant brain failure-free survival seen across multiple primary cancer subgroups, in the absence of active extracranial disease, may be a worthwhile treatment.

There are several limitations to the current study. While it is among the largest single institution datasets for brain metastases treated with SRS, its retrospective nature limits its interpretation to hypothesis-generation. There is a possibility for patient selection bias as patients with improved performance status may be more likely to receive targeted systemic therapy. Conversely, patients receiving targeted agents in the present study actually had a greater extracranial disease burden (43% vs. 32% widespread disease, *p* = 0.02) compared to those not receiving targeted agents. The heterogeneity of targeted agents and molecular targets across multiple tumor subtypes did not provide this dataset with sufficient power to stratify the analysis by specific targeted agents. In spite of its limitations, the results of the current study have wide implications for future prospective trials and the use of targeted agents in patients with brain metastases in order to help improve the therapeutic ratio of SRS, and possibly prevent the development of new brain metastases.

## MATERIALS AND METHODS

### Data source and acquisition

The study cohort was derived from the Wake Forest Gamma Knife database. This database included patients seen between January 2000 and December 2013 (737 patients) who underwent upfront SRS without whole brain radiation treatment (WBRT) for brain metastases. Patients who had previously received WBRT for brain metastases were excluded from the database since this treatment affects outcomes being measured in the study. Patients with brain metastases from sarcoma, ovarian cancer and head and neck cancers were also excluded because these patients represented such a small minority that the numbers were insufficient yield meaningful statistical conclusions. This study was approved by the Wake Forest School of Medicine Institutional Review Board and patient characteristics and treatment outcomes were determined using patients’ electronic medical records. Patient pre-treatment characteristics are shown in Table 2.

**Table 2:** Baseline characteristics

No. of Patients (%)			
Characteristic	SRS without Targeted Agents *N* = 570	SRS with Targeted Agents *N* = 167	*P*
Age at treatment			
Median (Min, Max)	63.0 (5.0, 91.0)	58.0 (21.0, 87.0)	0.002*
Gender			0.17
Women	256 (44.9%)	85 (50.9%)	
Men	314 (55.1%)	82 (49.1%)	
Primary Site of Brain Metastasis			0.0002
Lung	294 (51.6%)	70 (41.9%)	
Breast	64 (11.2%)	38 (22.8%)	
Renal/RCC	44 (7.7%)	24 (14.4%)	
Melanoma	97 (17.0%)	20 (12.0%)	
Colon	30 (5.3%)	10 (6.0%)	
Esophagus	15 (2.6%)	2 (1.2%)	
Other	26 (4.6%)	3 (1.8%)	
Histology			< 0.0001
Adenocarcinoma	213 (37.4%)	62 (37.1%)	
Squamous cell	54 (9.5%)	8 (4.8%)	
Adenosquamous	5 (0.9%)	0 (0.0%)	
Large cell NE	7 (1.2%)	0 (0.0%)	
Non-small cell lung NOS	47 (8.2%)	9 (5.4%)	
Her2-positive	18 (3.2%)	28 (16.8%)	
Her2-negative	38 (6.7%)	10 (6.0%)	
Breast other	7 (1.2%)	0 (0.0%)	
RCC	44 (7.7%)	24 (14.4%)	
Melanoma	97 (17.0%)	20 (12.0%)	
Other	40 (7.0%)	6 (3.6%)	
Number of Brain Metastases			0.23
1	299 (52.5%)	74 (44.3%)	
2	129 (22.6%)	40 (24.0%)	
3	70 (12.3%)	24 (14.4%)	
4 +	72 (12.6%)	29 (17.4%)	
Disease Burden^a^			0.02
None	98 (17.2%)	31 (18.6%)	
Oligometastatic	251 (44.0%)	54 (32.3%)	
Widespread	180 (31.6%)	72 (43.1%)	
Unknown	41 (7.2%)	10 (6.0%)	
Extracranial Disease			0.16
Stable	298 (52.4%)	101 (60.5%)	
Progressive	210 (36.9%)	53 (31.7%)	
Unknown	61 (10.7%)	13 (7.8%)	
DS-GPA mean (SD)	2.0 (0.9)	2.0 (0.9)	0.92
Number of courses of chemo			< 0.00001
0	442 (77.5%)	104 (62.2%)	
1	86 (15.1%)	14 (8.3%)	
2	20 (3.5%)	17 (10.2%)	
3	13 (2.3%)	17 (10.2%)	
4 +	9 (1.6%)	15 (8.9%)	
Margin Dose			
Median (IQR)	18.0 (17.0, 22.0)	20.0 (18.0, 21.0)	*0.2067
SRS Treatment Date mean	Feb 2008	May 2009	< 0.0001

Abbreviations: IQR, Interquartile range; RCC, Renal Cell Carcinoma; Large Cell NE, Large cell neuroendocrine; NOS, not otherwise specified; DS-GPA, Disease Specific Graded Prognostic Assessment.

a Disease burden was defined as none, unknown, oligometastatic (≤ 5 extracranial metastases), or widespread (≥ 5 extracranial metastases).

Patient factors including age, histology, disease-specific Graded Prognostic Assessment (ds-GPA), status of extracranial disease, and number of prior lines of systemic therapy were all determined from the electronic medical records. The ds-GPA class was defined as previously reported by Sperduto et al. [13]. The status of extracranial was categorized as “none”, “stable” or “progressive”. The extent of extracranial disease was characterized as none, oligometastatic, or widespread. Oligometastatic disease was defined as ≤ 5 non-brain metastases without diffuse involvement of any one organ. Widespread metastatic disease included patients with > 5 metastases or diffuse distant organ involvement.

### Endpoint definitions

Patients were followed clinically and with MRI at 4–8 weeks after primary radiosurgery. If there were no sign of treatment failure at this interval, clinical evaluation and MRI were conducted approximately every 3 months. Local failure was defined as tumor recurrence within the prior radiosurgical treatment volume. Local failure was determined via surgical pathology or imaging evidence of a 25% increase in area of enhancement on an axial MRI slice along with increased perfusion on perfusion-weighted imaging. Local failures were treated with surgical resection, whole brain irradiation, or observation depending on patient health status, the status of extracranial cancer, and physician discretion. Distant brain failure was defined as a new metastasis on follow-up imaging found outside the initial radiosurgical treatment volume. Distant brain failures were generally treated with further SRS, and WBRT was generally reserved for 5 + total brain metastases over time or short-interval distant failures. Neurological death was defined in the same manner as Patchell et al. [14].

### Radiosurgical technique

Patients were treated with Leksell Model B, C, or Perfexion units (Elekta AB). Prior to radiosurgery, patients underwent a high-resolution MRI of the brain. Treatment planning was performed using the Leksell GammaPlan Treatment Planning System (Elekta AB). A median dose of 20 Gy prescribed to the 50% isodose line was prescribed. Prescription dose was determined based on the guidelines previously published by Shaw et al. [15].

### Use of targeted agents

Targeted agents were generally used at the discretion of the treating medical oncologist. A targeted agent was defined as a systemic drug that inhibits a specific pathway(s) known to drive cancer growth. Selection of targeted agents by the medical oncologists was based on standard treatment algorithms specific to the cancer type of each patient. For example, agents targeting the human epidermal growth factor receptor (HER2), del 19, or L858R activating mutations in EGFR and the anaplastic lymphoma kinase pathway were used in these specific subgroups of breast and non-small cell lung cancer, respectively. BRAF inhibitors were utilized in melanoma as well as ipilumumab (Yervoy), which targets CTLA-4, a protein receptor that downregulates the immune system. Targeted agents utilized for renal cell carcinoma included tyrosine kinase inhibitors, mTOR inhibitors, or bevacizumab (Avastin). Wild type colorectal cancer patients often received agents that targeted EGFR such as cetuximab (Erbitux). Cytotoxic chemotherapeutic agents, those that more indiscriminately kill rapidly dividing cells, were not considered targeted agents in this study. Examples of cytotoxic chemotherapy include doxorubicin (Adriamycin) for breast cancer, pemetrexed (Alimta) for lung cancer, temozolomide (Temodar) for melanoma and capecitabine (Xeloda) for colorectal cancer.

### Statistics

Descriptive statistics were generated for the sample (*n* = 737) by targeted agent status. Patients were assigned to the cohort receiving targeted agents if they received a targeted agent either concurrently with SRS or within 1 month after completion of SRS. Differences between targeted agent status were determined using chi-squared tests for categorical characteristics and Kruskal-Wallis tests for age and margin dose due to the skewed distributions of these continuous measures. Kaplan-Meier plots and log-rank tests were used to compare the targeted agent status for overall survival, time to local failure, time to distant failure, and time to WBRT. These plots and tests were done on the whole sample as well as by primary site. A Cox proportional hazards model was created for the overall survival outcome using the predictors. From this model, hazard ratios, 95 percent confidence intervals were estimated. An alpha level of 0.05 was used to determine significance for all tests. All analyses were conducted using SAS 9.4 (SAS Institute, Cary, NC).

## CONCLUSIONS

Targeted agent use after SRS appears to significantly improve overall survival, local control, and the likelihood of distant brain failure. If these findings are prospectively validated, they would potentially provide new indications for targeted agent use in the setting of brain metastases receiving SRS.
